# Origins of the *Xylella fastidiosa* Prophage-Like Regions and Their Impact in Genome Differentiation

**DOI:** 10.1371/journal.pone.0004059

**Published:** 2008-12-31

**Authors:** Alessandro de Mello Varani, Rangel Celso Souza, Helder I. Nakaya, Wanessa Cristina de Lima, Luiz Gonzaga Paula de Almeida, Elliot Watanabe Kitajima, Jianchi Chen, Edwin Civerolo, Ana Tereza Ribeiro Vasconcelos, Marie-Anne Van Sluys

**Affiliations:** 1 Genome and Transposable Elements Laboratory (GaTE Lab), Departamento de Botânica, Instituto de Biociências, Universidade de São Paulo, São Paulo, São Paulo, Brazil; 2 Laboratório de Bioinformática (LABINFO), Laboratório Nacional de Computação Científica, Petrópolis, Rio de Janeiro, Brazil; 3 Emory Vaccine Center, Yerkes National Primate Research Center, Atlanta, Georgia, United States of America; 4 Escola Superior de Agricultura Luiz de Queiroz (ESALQ), Universidade de São Paulo, São Paulo, São Paulo, Brazil; 5 United States Department of Agriculture, Agricultural Research Service, San Joaquin Valley Agricultural Sciences Center, Parlier, California, United States of America; Massachusetts General Hospital, United States of America

## Abstract

*Xylella fastidiosa* is a Gram negative plant pathogen causing many economically important diseases, and analyses of completely sequenced *X. fastidiosa* genome strains allowed the identification of many prophage-like elements and possibly phage remnants, accounting for up to 15% of the genome composition. To better evaluate the recent evolution of the *X. fastidiosa* chromosome backbone among distinct pathovars, the number and location of prophage-like regions on two finished genomes (9a5c and Temecula1), and in two candidate molecules (Ann1 and Dixon) were assessed. Based on comparative best bidirectional hit analyses, the majority (51%) of the predicted genes in the *X. fastidiosa* prophage-like regions are related to structural phage genes belonging to the Siphoviridae family. Electron micrograph reveals the existence of putative viral particles with similar morphology to lambda phages in the bacterial cell *in planta*. Moreover, analysis of microarray data indicates that 9a5c strain cultivated under stress conditions presents enhanced expression of phage anti-repressor genes, suggesting switches from lysogenic to lytic cycle of phages under stress-induced situations. Furthermore, virulence-associated proteins and toxins are found within these prophage-like elements, thus suggesting an important role in host adaptation. Finally, clustering analyses of phage integrase genes based on multiple alignment patterns reveal they group in five lineages, all possessing a tyrosine recombinase catalytic domain, and phylogenetically close to other integrases found in phages that are genetic mosaics and able to perform generalized and specialized transduction. Integration sites and tRNA association is also evidenced. In summary, we present comparative and experimental evidence supporting the association and contribution of phage activity on the differentiation of *Xylella* genomes.

## Introduction

*Xylella fastidiosa* is a gram-negative gamma-proteobacterium known to cause several economically important diseases in cultivated crops and many other plant species. The strain 9a5c (Xf-CVC) was the first plant pathogen whose genome was completely sequenced [1]. This was followed by the publication of draft sequences from the gapped-genomes of strains Dixon (Xf-ALS) and Ann1 (Xf-OLS) [2] and the complete genome of the Pierce Disease associated Temecula-1 strain (Xf-PD) [3]. Genomic analyses in different *Xylella* strains reveal interesting biological and evolutionary aspects regarding genome structure and gene content. Previous studies demonstrated that 98% of the Xf-PD genes are shared with Xf-CVC, with an average amino acid identity (considering only the coding regions) of 95.7%, and the main differences are from bacteriophage-derived regions. These bacteriophage-derived regions are responsible for chromosomal rearrangements and deletions in *X. fastidiosa* strains, thus playing a decisive role on the genome evolution of this plant pathogen [1], [3].

Recently published work demonstrate that virus particles, including bacteriophages, appear to be strikingly abundant, with a typical estimated concentration of 10^7^ particles/ml in coastal sea water and even higher in some other habitats, such as freshwater ponds [4]. Based on these data, it is proposed that these particles represent the most abundant biological form on Earth [5] and potentially an efficient vehicle for lateral gene transfer (LGT). A large body of sequence data is generated by projects of bacteriophage genome sequencing, and almost 500 phage genomes have been determined and deposited in Genbank, and together with metagenomics studies (mainly from environmental samples), indicate a broad genetic diversity, representing the largest reservoir of sequence information in the biosphere [6], [7]. Moreover completely sequenced phage genomes have a high degree of mosaicism probably derived from extensive horizontal genetic exchange occurring over perhaps as many as 3 billion years [8]–[10]. Furthermore, bacteriophages have a central role in the evolution of their bacterial hosts and the emergence of new pathogens, by moving genes from host to host as a mechanism that generate gene and genome diversity, thus constituting, in many bacterial species, a substantial part of acquired DNA [5], [11]. In some instances, lysogenic conversion of phages are of selective advantage to the bacterial host, as they can dramatically affect host phenotype [12].

To accomplish integration, temperate bacteriophages encode a phage integrase enzyme that mediates recombination between short sequences of phage DNA, the phage attachment site *attP*, and a short sequence of bacterial DNA, the bacterial attachment site *attB*. Phage integrases all fall into a category of enzymes known as site-specific recombinases [13]. Each phage integrase recognizes distinct *attB* sequences and are grouped into two major families, based on their mode of catalysis: the tyrosine and the serine recombinases [14]. At least 75% of the phage tyrosine recombinases use tRNA sequences as attachment sites in bacteria, indicating that tRNAs are directly involved in the phage acquisition process [15].

In this work, the genomes of four *X. fastidiosa* strains, Xf-CVC, Xf-PD, Xf-OLS, and Xf-ALS, were compared with regard to their prophage content and respective predicted integrase genes. A total of 56 predicted integrases were identified, and network analysis and phylogenetic reconstructions support the existence of five major lineages related to known bacteriophages that infect gamma and beta-proteobacteria. By Bidirectional Best Hit (BBH) analysis (against 402 bacteriophage genomes), the integrases were all found to be associated mainly to phages containing structural genes of Caudovirales viruses. *In silico* gene expression analysis of Xf-CVC prophage-like regions reveals these prophages are probably actively transcribed, and this finding is supported by the presence of putative phage-like particles in *Xylella* cells both *in planta* (almond petiole and hibiscus leaves) and *in vitro* [16]. Comparative studies conducted on the structure of the prophage regions and their relative genomic positions strengthen their impact in the genome organization and differentiation of these closely related *X. fastidiosa* strains.

## Results

### 1. Prophage-like elements: organization, diversity and comparative analysis

#### a) Identification of prophage-like regions

Identification and definition of prophage-like elements is not trivial task, but an empirical approach that needs a lot of insight [17].In the previously reported Xf-ALS and Xf-OLS assemblies [2], prophage-like elements were not identified, and candidate molecules were not determined for each strain. A specific strategy to resolve the assembly of phage-related regions was developed which generated candidate molecules for each strain, thus making possible the identification of prophage-like elements and phage remnants (data not shown) In the same way, identification of all predicted integrase genes and prophage-like regions were carried in Xf-CVC 9a5c and Xf-PD Temecula strain genomes.

All four *X. fastidiosa* genomes were scanned for the presence of predicted integrases associated to clusters of genes related to phages. Regions encompassing more than 10,000 bases and at least 80% of phage-related genes in their constitution were defined as prophage-like regions; the smaller ones were defined as prophage remnants. This strategy enabled the identification of 46 chromosome fragments in *X. fastidiosa* genomes predicted to be descended from ancestral invading bacteriophages (Table 1). The sum of all elected regions represents 1,300,341 bp of the *X. fastidiosa* chromosomal backbone as being of bacteriophage origin. The CVC strain contains six prophage-like regions and five phage remnant regions. In previously published work a conservative approach was utilized and defined four prophage-like regions named xfp1–xfp4 [1]. The PD strain contains eight prophage-like regions and two small phage remnant regions already reported by Van Sluys and colleagues [3]. The OLS strain candidate molecule contains ten prophage-like regions and only one phage remnant region. Finally, the Xf-ALS strain candidate molecule contains eleven prophage-like regions and three phage remnants.

**Table 1:** General genomic features of each phage-related and GI element identified in four *Xylella* strains.

	Length (bp)	# ORFs	Integrase a	Associated tRNA b	Status
**XF-CVC**					
**xfp1**	42,178	54	(1) Full-length	VAL	Probable complete
**xfp2**	43,708	58	(1) Full-length / (1) Fragment	VAL (frag)	Probable complete
**xfp3**	26,940	44	(1) FS / SCF	-	Defective
**xfp4**	45,930	69	(1) Full-length	ARG	Probable defective
**xfp5**	18,184	31	(1) FS / SCF	CYS	Defective
**xfp6**	43,585	57	(1) Full-length	ASN	Probable complete
**giCVC**	67,058	78	(1) Full-length / (2) Fragments	GLY	Genomic Island
**cvc-r -1**	14,946	8	(1) Full-length	SER	Phage remnant
**cvc-r -2**	1,682	1	(1) Fragment	VAL	Phage remnant
**cvc-r -3**	6,919	13	(1) Fragment	LYS	Phage remnant
**cvc-r -4**	14,561	20	(1) Full-length	GLY	Phage remnant
**cvc-r -5**	16,819	22	-	LYS	Phage remnant
**XF-PD**					
**xpd1**	55,498	76	(2) Full-length / (1) Fragment	-	Probable complete
**xdp2** *	62,087	85	(2) Full-length	-	Probable complete
**xpd3**	13,911	22	(1) Full-length	-	Probable defective
**xpd4**	16,295	24	(1) Full-length	-	Probable defective
**xpd5**	24,192	41	-	LYS	Defective
**xpd6**	27,651	45	(1) FS / SCF	GLY	Defective
**xpd7**	17,795	29	(1) FS / SCF	VAL	Defective
**xpd8**	15,302	21	(1) FS / SCF	ASN	Defective
**pd-r-1**	6,613	8	(1) Full-length	CYS	Phage remnant
**pd-r-2**	407	1	(1) Fragment	VAL	Phage remnant
**XF-OLS**					
**xop1**	17,201	24	-	-	Defective
**xop2**	32,742	39	(1) Full-length	-	Probable complete
**xop3**	41,771	60	(1) Full-length	-	Probable complete
**xop4**	22,988	40	(1) Full-length	-	Probable defective
**xop5**	17,738	36	(1) Full-length	ASN	Defective
**xop6**	41,004	59	(2) Full-length	LYS	Probable complete
**xop7**	38,303	63	(1) FS / SCF / (1) Fragment	THR	Defective
**xop8**	40,886	72	(1) Full-length / (1) FS / SCF	-	Probable defective
**xop9**	43,551	66	(1) Full-length	VAL	Probable defective
**xop10**	32,915	65	(1) Full-length	GLY	Probable defective
**ol-r-1**	10,484	7	(1) FS / SCF	CYS	Phage remnant
**XF-ALS**					
**xap1**	41,622	62	(1) Full-length / (1) Fragment	VAL (frag)	Probable complete
**xap2**	22,978	29	(1) Full-length	CYS	Probable defective
**xap3**	48,027	76	(1) Full-length	GLY	Probable complete
**xap4**	20,150	32	(2) Full-length	ASN (frag)	Probable defective
**xap5**	37,661	59	(1) Full-length / (1) Fragment	VAL	Probable complete
**xap6**	39,002	54	(1) Full-length	-	Probable complete
**xap7**	26,309	42	(1) Full-length	-	Probable complete
**xap8**	42,407	63	-	-	Defective
**xap9**	45,251	73	(1) Full-length / (1) FS /SCF	VAL	Probable complete
**xap10**	18,200	27	(1) Fragment	LYS (frag)	Defective
**xap11**	14,923	21	(1) Fragment	LYS	Defective
**al-r-1**	2,740	2	(1) Fragment	GLY	Phage remnant
**al-r-2**	10,691	14	(1) FS /SCF	-	Phage remnant
**al-r-3**	8,536	10	(1) Full-length	SER	Phage remnant

a FS / SCF = frameshift or stop codon in frame.

b frag = tRNA fragment.

* giPD is located within xpd2.

Almost all of these regions carry integrases, and by comparative analyses of the open reading frames (ORFs) composition, it is possible to infer the candidates to be a probable complete or a defective prophage, for each strain (Table 1). The prophage-like and phage remnant regions span 342,510 bp (12.53%), 239,751 bp (9.50%), 339,583 bp (12.97%) and 378,497 bp (14.39%) of CVC, PD, OLS and XF-ALS strains, respectively. The average length is 32 kb for prophage-like and 7.5 kb for phage remnant regions.

Most of the prophage-like regions (60%) are localized between the position 900 kb and 1,800 kb of the chromosome (position 1 being the putative origin of replication, in clockwise orientation positioned at *dna*A gene), some of them positioned near the putative terminus of replication (as determined by GC skew analysis). This particular distribution is suggestive that these prophage elements may represent recent acquisition in the *X. fastidiosa* genome probably relating to the moment in the cell cycle that insertion occurs as previously demonstrated for other prokaryotic genomes (20).

**Figure 1:**
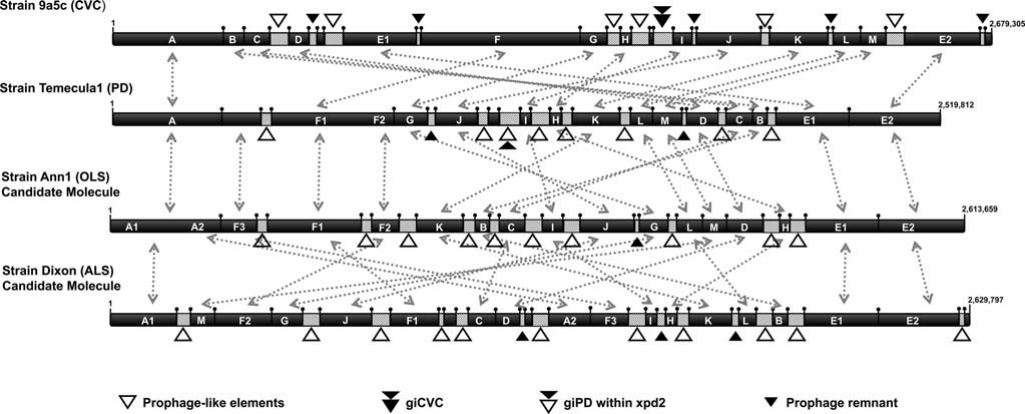
Schematic representation of chromosome alignment of four *X. fastidiosa* strains. The genomes of four *X. fastidiosa* strains (9a5c/Xf-CVC, Temecula1/Xf-PD, Dixon/Xf-ALS and Ann1/Xf-OLS) were aligned starting from the predicted origin of replication (number 1 arrow), to the end of the genome (indicated by the arrow and number with the specific length of each strain). The black bars represent the chromosome of each strain; the letters (A–M) inside each bar depict the chromosome backbone, indicating the relative position and size of collinear chromosomal regions. Rearrangements between each strain are shown by grey dotted arrows. White triangles illustrate the position of prophage-like and genomic island regions; prophage remnants are indicated by black triangles. Each prophage or genomic island region indicated by a triangle is represented by a grey bar inside the chromosome representation (the size is relative to the length of each mobile genetic element).

In addition, the majority of the rearrangements observed among the four genomes are concentrated in this terminal region. Alignment of the two chromosomes (Xf-CVC and Xf-PD) and the two candidate molecules (Xf-ALS and Xf-OLS), starting from the putative origin of replication, reveals at least 16 chromosomal regions in the four genomes that are translocated and/or inverted. The Xf-ALS strain presents 16 disruptions in its candidate molecule compared to the other three strains, followed by Xf-CVC strain with 14, and 13 chromosomal breaks in Xf-OLS and Xf-PD strains, suggesting that Xf-ALS strain is the most divergent in terms of genome structure and this divergence is directly associated to phage insertions. The association of phage related regions with breaks in chromosomal colinearity has been previously described when comparing Xf-CVC and Xf-PD genomes [3] and is now expanded to the other *X. fastidiosa* sequenced strains. Xf-PD and Xf-OLS strains display only 7 chromosomal breaks between each other, supporting these strains as the more recently diverged. In all cases, rearrangements were caused by phage insertion, as each break is bordered by a prophage-like or a phage remnant region, suggesting an important role of the phages in the genome organization of *X. fastidiosa* genomes (Figure 1). Taken together, these results helped to strengthen the association of the prophage-like regions with the differentiation of *X. fastidiosa* strains chromosomes.

#### b) Comparisons among prophage-like regions

This analysis enables us to trace a possible evolutive scenario for each group of prophages-like elements. This analysis enables to hypothesize the most ancient insertions relative to the most recent ones. Firstly we report the elements inserted in the same genome context in different strains, indicating preferred sites of insertion; that, the ones related to common ancestral events.

There is only one prophage insertion shared by the four strains with mostly the same gene content and genome borders: xpd6, xop10, cvc-r4 and al-r2. These regions possess the same upstream genome border located near an epsP synthase, and the downstream border near a tonB-dependent receptor, except for the xop10 element, where the downstream gene is located close to a methyltransferase. The cvc-r4 and al-r2 remnant regions appear to be degenerate regions that originated from a xop10-like ancestor. Region xpd6 appears to be a degenerate form of xop10, mainly by the presence of a frameshift in the xpd6 integrase, suggesting that xop10 might be the closest to the common ancestor of this group. The xpd6 and xop10 regions share 76.1% nucleotide identity and carry 49% of putative non-essential phage ORFs, 45% of hypothetical ORFs and only 6% of essential phage ORFs. The gene content of these regions includes a copy of virulence-associated protein I and a hicA/hicB toxin-anti-toxin system. Neither of these regions contains structural phage genes. The xpd6, cvc-r4 and xap10 elements have a tRNA-GLY in their constitution, suggesting a mechanism of acquisition of this tRNA by transduction.

Another site of insertion is shared by three different strains, and it involves the remnants pd-r1, ol-r1, and the xap2 element. They are inserted between a fumarate hydratase and glucose inhibited division protein, in the vicinity of a tRNA-CYS. These three regions are most probably defective prophage in the process of genome decay, and pd-r1 and ol-r1 appear to be degenerated versions of xap2.

In these two cases, the analysis strongly suggests an evolutionary mechanism of negative pressure in order to delete or fully inactivate these regions of the chromosome, in accordance with to previous studies in others prophage regions [17], [18]. Deletions and mutations occur in these regions especially in the remnant regions, independent of the boundaries of the genome core, suggesting genome decay in prophage-like and remnant regions. The remaining prophage regions are inserted in regions shared by only two strains, or are in unique positions in a specific strain representing late infection events. There are seven sites of insertion shared by prophage elements from two different strains.

Seven prophage-like elements, xfp1, xfp2, xpd1, xop3, xap1, xap5 and xap6 are involved in large genome rearrangements and share at least 80% of nucleotide identity. These appear to be complete phages. Gene order and orientation is highly conserved among all the seven elements, the integrases being followed by non-structural (as DNA helicase, DNA polymerase, primase, and phage repressor and anti-repressor genes) and structural genes with both classes separated by an endolysin gene. It is interesting to note that the DNA-packaging and head genes resemble in organization and sequence the lambda-like phages, and the baseplate, tail, and tail fibers genes (gpV, gpW, gpJ, gpI, gpU, gpX and gpD) resemble the P2-like phages. This architecture suggests a hybrid phage (as previously observed for xfp1 and xfp2 [17]), split in two by the presence of a system of toxin and anti-toxin genes in xfp1, xpd1, xop3, xap1, xap5, and xap6 (in the same position, the xfp2 element has a transcriptional regulator). The non-structural genes are composed of the integrase followed by polymerase, repressor, anti-repressor and the primase, resembling in organization and sequence the APSE-1 phage that infects *Acyrthosiphon pisum* [19].

Another class of related prophage regions display 80% nucleotide identity but it is restricted to the structural genes (xfp3, xfp4, xpd2, xop9, xap3, xap7 and xap9) The endolysin gene separates the non-structural genes from the structural ones, resembling in organization and sequence the prophage 4 that infect *Listeria innocua* (derived from the phage Sfi11), and the phage AaΦ23 from *Actinobacillus actinomycetemcomitans*, generally associated to human oral infections [20].

The fact that these elements are inserted at different genomic positions in the four strains, and that they share extensive sequence similarity suggest recent and independent acquisitions. This similarity also implies that these bacteriophages are frequent entities in the environment including the riparian vegetation, insect vector or any of the infected plants.

The hybrid origin of the prophage regions is probably the result of illegitimate recombination in the process of the horizontal genetic exchange, as observed in Mycobacteriophages by Pedulla et al [7] and in *Escherichia coli*, *Vibrio cholerae* and *Staphylococcus aureus* by Boyd et al [21]. On another note, the larger number of genomic rearrangements of Xf-CVC and Xf-ALS compared to Xf-PD and Xf-OLS may result from the presence of long repeats in the same chromosome which can act as rearrangement sites by homologous recombination. Along this trend, the duplicated prophage elements, [xfp1 e xfp2], [xfp3 e xfp4] in Xf-CVC, and [xap1, xap5 e xap6]; [xap3, xap7 e xap9] in Xf-ALS, would be responsible by the higher number of rearrangements in these strains providing indirect evidences of the impact of these elements in re-structuring the bacterial genome backbone.

#### c) Comparative Analysis of the Genome Content of the Prophage-like Regions

From a total of 1,728 prophage-like genes in the four strains, 1,388 (80.5%) belong to 290 different best bidirectional hit (BBH) clusters, while 339 (19.5%) are not present in any BBH cluster. The latter group represents strain-specific prophage-like genes. In this specific group, 66 (19.5%) are ORFs with putative functions related to essential phage genes, and 28 (8.25%) to non-essential phages genes (potentially involved in yet-to-be-established phage functions), while the remaining 245 ORFs (72.25%) are hypothetical or conserved hypothetical genes, representing an abundant number of ORFs that can be related to genomic differentiation.

The most interesting cases of prophage-like ORFs in BBH clusters, and potentially related to bacterial pathogenicity, are the putative phage-related PI protein (Zonular occludens toxin- like protein) present in xop7, xap10 and xpd5 ; and the virulence-associated protein E, present in xfp5, xfp6, xpd8, xop6, xap8. The products of these ORFs may be related to interactions between the plant and the bacteria. Phage PI protein (zot) is required for phage assembly [22] and the copies found in *X. fastidiosa* share 55% identity with orthologs found in the filamentous phage phiLf of *Xanthomonas campestris* pv. v*esicatoria* and phage phiSMA9 of *Stenotrophomonas maltophilia*, and with less than 30% identity to orthologs of *X. campestris* pv. *campestris* and RSM1 phage of *Ralstonia solanacearum* and *R. pickettii* genome. These organisms are necrogenic plant pathogens, except *Stenotrophomonas*, a human pathogen also able to colonize diverse plants, especially those in the *Brassicaceae* family [23]. The hypothesis is that zot protein was acquired by the few clinical *Stenotrophomonas* strains only recently after infection by a filamentous phage, which probably came after certain changes in its adsorption protein from plant-pathogens [24].

A group of toxin and anti-toxin proteins in prophage-like regions (higA/higB and relE/relB) was also found. These proteins are very common in plasmids, where they increase effective stability [25], and in bacterial chromosomes (probably by phage acquisition), where they are related to stabilization of phage genomes in the host chromosome by reduction of the effective deletion rate or, contrarily, to anti-phage functions. In this case, the phage can interfere with host transcription and translation, activating addictive systems, which would then limit phage production [26].

Furthermore, the group of specific ORFs (belonging to no BBH cluster) related to non-essential phage functions have some interesting components: (a) virulence-associated protein in xfp3 (VapB-like), exclusive for the Xf-CVC strain; (b) modification methylase NspV and restriction NspV enzymes in xpd8; (c) the restriction enzyme NgoMIV and modification methylase NgoMIV in xap4; and (d) virulence-associated protein I in xop10, (and a truncated copy in xpd6, with only 53% of the length of the original). All these ORFs may be involved in interactions between plant and bacteria (a and d), or between bacterial and phage genomes (b and c). On the other hand, these ORFs do not have BBH pairs against the 402 phage genomes, suggesting they are not necessary for phage biology, but with exclusive roles in each *Xylella* strain.

#### d) Enrichment of Xf-CVC prophage-like ORFs in different Xylella strains

A previous study of microarray hybridization was carried out in order to compare six different citrus-associated *Xylella* strains from symptomatic and asymptomatic plants isolated in South American (using as reference the 9a5c strain genome) (for further information, refer to da Silva et al [27] and GEO database, entry GSE8493). Briefly, all six strains were from a culture collection and collected from sweet orange trees: four (56a, 9.12c, 187b, and 36f) were obtained from CVC-affected trees, and represent the most prevalent haplotypes, while strain CV21 was obtained from a non-symptomatic tree from the same orchard; on the other hand, strain Fb7 was obtained from a plant with symptoms of a CVC-similar disease (pecosita).

In the present study, we re-analyzed these microarray series focusing on the prophage-like regions. The results exhibit an enrichment of prophage-like ORFs of 22% against 7.6% of the remaining ORFs (those outside prophage regions, and representing the core genome) (Table 2). High copy numbers of xfp5 and xfp6 elements are present in all strains, and these regions possess one copy of the virulence-associated protein E. Although element xfp5 appears to be a defective prophage in Xf-CVC strain, the enrichment observed in other strains may suggest that this region has important roles in other strains. The xfp1 and xfp2 elements are probable complete prophages with ORF composition resembling a hybrid lambda and T4 phage, and they are present in high copy in 56a strain. These data suggest that these elements are under a lytic life cycle and may represent the real-time action of phages in these strains.

**Table 2:** Diversity of Xf-CVC phages in six different strains of *X. fastidiosa* by hybridization analysis*.

XF-CVC Element	Xylella strains					
	187b	36f	56a	9.12c	Cv21	Fb7
**xfp1**	Equal	Equal	Higher (>50%)	Equal	Equal	Equal
**xfp2**	Equal	Equal	Higher (>40%)	Higher (>40%)	Equal	Equal
**xfp3**	Equal	Equal	Equal	Equal	Equal	Equal
**xfp4**	Equal	Equal	Equal (20% absents)	Equal (20% absents)	Equal (20% absents)	Equal
**xfp5**	Higher (>50%)	Higher (>40%)	Higher (>40%)	Higher (>40%)	Higher (>40%)	Equal
**xfp6**	Higher (>50%)	Higher (>40%)	Higher (>50%)	Higher (>50%)	Higher (>50%)	Equal
**cvc-r1**	Higher (>50%)	Equal	Equal	Equal	Equal	Equal
**cvc-r2**	Equal	Equal	Equal	Equal	Equal	Equal
**cvc-r3**	Equal	Equal	Equal	Equal	Equal	Equal
**cvc-r4**	Equal	Equal	Equal	Equal	Equal	Equal
**cvc-r5**	Equal	Equal	Equal (50% absents)	Equal (50% absents)	Equal (30% absents)	Equal

* Presence is given in terms of number of copies of each ORF within phages (data extracted from GEO database GSE8493).

Region xfp4 is present in low copy number in both symptomatic and non-symptomatic strains (56a, 9.12c and CV21) and in equal copy numbers in the other strains. The principal feature of this region is the presence of three systems of toxin and anti-toxin genes. Despite the xfp4 appearing to be a defective prophage, this element apparently is not active at least in strains 56a, 9.12c and CV21. This supports the idea that this element is stable, under lysogenic state, being subject of genome decay or stabilization in the host by a selective negative pressure. These findings indicate that: (1) enrichment in the number of copies of ORFs in prophage-like regions compared to the core genome ORFs in different strains, (2) the different prophage-like regions have diverse hybridization profiles, and (3) a lytic cycle activity with the formation of new phage particles. Thus, element xfp4 may play an important, but different, role in different *Xylella* strains.

#### e) Prophage-like regions: patterns of expression in stress conditions

Previous microarray analyses of Xf-CVC strain 9a5c described the expression profile under stress conditions, particularly under heat shock conditions (for further details, refer to [28]–[30] and GEO database entries GSE6619, GSE4161, GSE3044 and GSE4960). These microarray series were re-analyzed with the focus on the prophage-like regions (Table 3).

**Table 3:** Phage-related genes differentially expressed under different stress conditions.

Prophage-like Element	ORF ID	Product	Expression in 3G10R a	Expression at 40°C b	Expression at 40°C c	Expression at 40°C d
**xfp1**	XF0678	phage-related integrase		↑		
	XF0684	phage-related antirepressor		↑	↑	↑
	XF0685	phage-related protein P50	↑	↑		
	XF0686	phage-related protein P51	↑	↑		
	XF0704	phage-related antirepressor	↑	↑	↑	
	XF0717	phage-related minor tail protein		↑	↑	
	XF0718	phage-related protein	↑	↑		
	XF0719	phage-related baseplate assembly protein V		↑	↑	
**xfp2**	XF2488	phage-related baseplate assembly protein J				↓
	XF2491	HTH-type transcriptional regulator			↑	
	XF2492	phage-realted baseplate assembly protein V			↑	
	XF2494	phage-related minor tail protein		↑	↑	
	XF2495	phage-related protein		↑	↑	
	XF2496	phage-related protein			↑	
	XF2511	phage-related repressor protein CI		↓	↓	
	XF2522	phage-related putative protein P51	↑	↑	↑	
	XF2523	phage-related putative protein P50		↑		
	XF2525	phage-related DNA polymerase (P45)		↑	↑	
	XF2526	phage-related putative protein P44		↓		
**xfp3**	XF1559	phage-related regulatory protein (antirepressor)	↑			
	XF1588	Virulence-associated protein		↑		
	XF1590	plasmid stabilization protein		↓		
	XF1598	phage-related protein		↑		
	XF1599	phage-related tail fiber protein			↓	
**xfp4**	XF1644	Single-stranded DNA-binding protein	↑			
	XF1645	phage-related antirepressor		↑	↑	
	XF1647	phage-related protein		↑		
	XF1663	phage-related antirepressor		↑	↑	
	XF1668	HicB-related protein		↑	↑	
	XF1686	phage-related protein		↑		
	XF1687	phage-related protein	↑			
	XF1696	Anti-toxin RelB protein			↑	
	XF1703	phage-related addiction module killer protein		↑		
	XF1706	phage-related long tail fiber protein	↑			
	XF1710	transcriptional regulator	↑			
**xfp5**	XF2110	DNA binding transcriptional regulator	↓			
	XF2115	phage-related protein	↑			
	XF2120	phage-related terminase protein				↓
	XF2121	virulence-associated protein E	↑			
	XF2122	DNA primase	↑			
	XF2129	phage-related protein		↑		
**xfp6**	XF0480	phage-related integrase	↑			
	XF0483	phage-related protein		↑		
	XF0487	Tfp pilus assembly protein, major pilin FimA/PilA	↑			
	XF0512	phage-related protein	↓			
	XF0535	Transposase, IS200/IS605 family	↓			
**cvc-r3**	XF2761	phage-related integrase (fragment)		↑		
**cvc-r4**	XF2298	Phosphotyrosine protein phosphatase			↓	
	XF2302	Glutamate-1-semialdehyde 2,1-aminomutase			↓	
	XF2305	Glyoxalase-like protein	↓			
**cvc-r5**	XF1859	Phage-related replication protein rstA	↑		↑	
	XF1864	phage-related protein		↑	↑	
	XF1869	phage-related protein	↑			

a Expression under different medium growth conditions (3G10R against PW) (data extracted from da Silva et al [27] and GEO database GSE6619).

b Expression under heat shock response, at 40°C, when compared to normal conditions of temperature (25°C) (data extracted from Koide et al [30] and GEO database GSE4161).

c Expression under heat shock response, at 40°C, when compared to normal conditions of temperature (29°C) (data extracted from Vencio et al [57] and GEO database GSE3044).

d Expression of mutant strain (rpoE) of the strain J1a12 (against 9a5c array), under heat shock response, at 40°C, when compared to normal conditions of temperature (25°C) (data extracted from da Silva Neto et al [28] and GEO database GSE4960).

Interestingly, all Xf-CVC prophage-like regions have genes differentially expressed when the bacteria are under stress conditions, and even in the phage remnants some genes are differentially regulated. Notably, among the differentially expressed genes, phage genes tend to be up-regulated genes (66%) more frequently than those that are down-regulated (34%). Anti-repressor genes are over-expressed (5 out of 6 genes), followed by genes involved in phage replication and structural genes, as well some integrases (xfp1, xfp6 and cvc-r3), while repressor genes are under-expressed. This suggests that under stress conditions the prophage-like regions are activated and may trigger induction of the lytic cycle, which ultimately results in the formation of virus-like particles (VLPs). Both switches from 25°C to 40°C and from 29°C to 40°C result in clear induction of gene expression of prophage genes which could be indicative that changes in the temperature in the orchard along the growing season of the plants could result in bursts of phage induction within the plant.

Along the line, but in a distinct relation, the combined analysis of the DNA-DNA hybridization array [27] and the expression array studies just presented [28]–[30] demonstrate that xfp5 and xfp6 are probably producing VLPs under normal growth conditions as would be xfp1 and xfp2. The two latter present induction of gene expression under stress condition and the anti repressor gene is induced under these conditions eventually resulting in an increase of VLP production. The biology of these changes in the pattern of phage-related regions unveils a possible association of these elements in the disease development. Also, as originally pointed by the authors [28], these prophage genes are probably under rpoE control as their induction is not detected when mutant strain is utilized.

It is also worth noting that genes not directly related to the phage structure, as anti-toxin and virulence-associated genes, are also induced under these conditions. For example, over-expression of the virulence protein present inside the xfp3 element, a protein that occurs exclusively in this element and strain, indicates a role in heat-shock conditions. Accordingly, the high number of hypothetical phage-related genes that are also differentially expressed suggests some important, yet unknown, roles for this class of genes.

**Figure 2:**
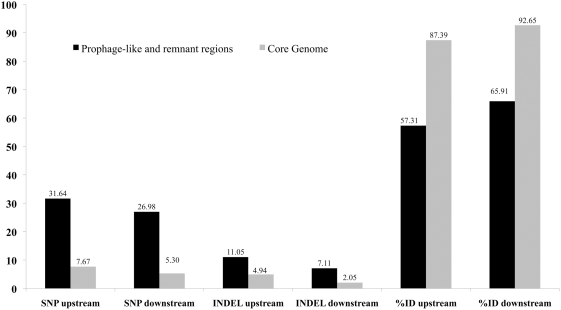
Analysis of up- and downstream regions in prophage-like ORFs. Proportion of single nucleotide polymorphisms (SNPs) and insertions and deletions (INDELs) as well the percentage of nucleotide identity (%ID) were analyzed comparing 250 bp up- and downstream regions of each prophage-like predicted ORF against the core genome (all remaining non-prophage ORFs in each genome). Results are represented by black (for the prophage-like ORFs) and grey (for the core genome ORFs) bars.

An extensive analysis of the 250-bp upstream and downstream regions of each prophage-like ORF (compared to all non prophage-like ORFs located in the rest of the genome, herein called core genome) reveals that the prophages and remnant regions have an increased number of single nucleotide polymorphism and insertions and deletions, as well a reduced percentage of overall nucleotide identity when compared to the core genome (Figure 2). The nucleotide identity among collinear up- and downstream regions in the core genome is higher than 87% while it ranges from 57% to 65% in the prophage-like regions. This suggests these latter regions are most probably evolving more rapidly and that similar genes may present distinct expression profile due to promoter region variation. These findings also suggest that these prophage-like elements are under an intense mechanism of recombination of similar phages, and that mechanism can be responsible for the generation of mosaic architecture in these regions.

#### f) Bacteriophage origin of the prophage-like regions

All prophage-like ORFs with putative functions related to structural phage genes (i.e., capsid, fiber, tail, scaffold, and baseplate) are grouped in BBH clusters. The phage family represented the most in these BBH pair groups of BBH is the Siphoviridae family (51%), followed for Myoviridae (32%) and Podoviridae (10%) families (7% of the BBH pairs are from unclassified Caudoviridae phages). The most important and studied phage within the Siphoviridae family is phage lambda, widely found in the chromosome of enterobacteria, where it plays diverse biological roles, most of them related to acquisition of virulence genes by the bacteria through LGT [31]. In a extensive review of morphology of phage particles carried out by Ackermann [32] the closest-related *Xanthomonas* genus have at least 35 types of tailed phages, where 25 (72%), 9 (25%) and 1 (3%) and are from Siphoviridae, Myoviridae and Podoviridae families respectively.

**Figure 3:**
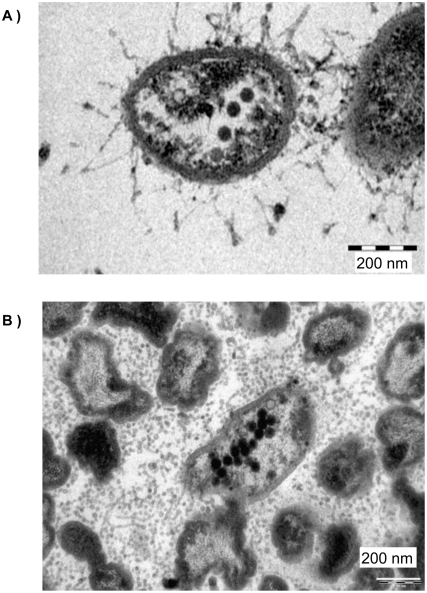
Phage-like particles viewed with transmission-electron micrographs of *Xylella fastidiosa* cells *in planta*. Putative icosahedral phage-like particles are present inside and outside the cell, which resemble a putative phage lambda-like particle. A. Almond petiole plant, B. Petiole of *Hibiscus* plant.

Moreover, viral particles with icosahedral lambda-like morphology within bacterial cells *in planta* were observed by transmission electron microscopy (Figure 3). This suggests the possibility of a common *Xylella* phage ancestor from the lambda phage family. The data suggests that the prophage-like elements present in *Xylella* genomes represent not only an ancient and stalled event that occurred in the genome before the differentiation of the strains, but a dynamic and real-time event by temperate phages dramatically shapes the genome. Putative *X. fastidiosa* phages could carry specialized components that may confer a level of specialization or advantage to their host, in a similar manner as in *E. coli*, *Streptococcus pyogenes* and *Staphylococcus aureus* [33]–[35].

### 2. Integrases: diversity, domains and site of integration associated with tRNAs

#### a) Identification, clusterization and domains of prophage-related integrases

Integrases are useful markers for identifying prophages and potential indicatives of LGT events in bacterial genomes [17]. They are also required to either establish or exit from a lysogenic state. All the integrases identified in the *Xylella* genomes belong to the family of tyrosine recombinases, with the potential catalytic signature identified (as proposed by [14]), and possessing protein domains related to the breaking and rejoining of single strands in pairs to form a Holliday junction intermediate. Almost all prophage-like regions (42 out of 46) in the four *X. fastidiosa* strains bear one or two putative integrase genes (Table 1). The integrases in *X. fastidiosa* occur in three distinct forms: (1) full-length gene; (2) ORF with a frameshift (FS) or a stop codon in the frame (SCF); or (3) small fragments (less than 150 residues). There are 33 full-length integrases: ten in the Xf-ALS, nine in the Xf-OLS, and seven each in the Xf-CVC and Xf-PD strains. Most of these are associated with the largest prophage-like regions and the genomic islands (giCVC and giPD). There are ten integrases with FS/SCF three each in the Xf- PD and Xf-OLS, and two each in the Xf-CVC and Xf-ALS strains, all associated to prophage-like regions with length less than 20 kb. Finally, there are 13 integrase fragments, with five each in the Xf-CVC and Xf-ALS, two in the Xf-PD, and one in the Xf-OLS strains, most of them associated with phage remnants.

There are three main relationships between the integrases and these prophage elements: (1) all the (potentially) complete and the largest prophage elements carry full-length integrases, (2) truncated integrases (with SCF/FS) are present in probable defective and smaller prophages and (3) fragments of integrases are found mainly in phage-remnants, while non-remnant regions always bear another full-length integrase when a fragment is present. These results suggest the existence of a selective negative pressure associated with the integrase inactivation with further genome decay of the most ancient prophage elements.

From the alignment with model tyrosine-recombinases, the conserved active residues R [212], K [225], H [308], R [311], H [333] and Y [342] (numbers within brackets refer to the model integrase from the lambda-phage integrase; see [14]), responsible for the catalytic activity, were identified at the C-terminal end of all full-length phage integrases. The exception is residue H [308], not found in the majority of the integrases. However, this residue is also absent from several other tyrosine-integrases (according to the model deposited on the Conserved Domain Database). Variation in spacing and amino acid sequence was observed from class to class. Nevertheless this motif is remarkably conserved (Figure S1). Moreover, all full-length integrases possess the protein domains related to lambda and P4 integrases.

The only exception is related to the integrases identified within the genomic islands giCVC and giPD that are present in the genomes of the Xf-CVC and Xf-PD strains, respectively [3]. These integrases do not possess the same catalytic signature and similarity of amino acid residues as with the other *Xylella* integrases associated with prophage-like regions. However, they have a characteristic protein domain from phage CP4 integrase.

#### b) Evolutionary Relations of the Integrases

**Figure 4:**
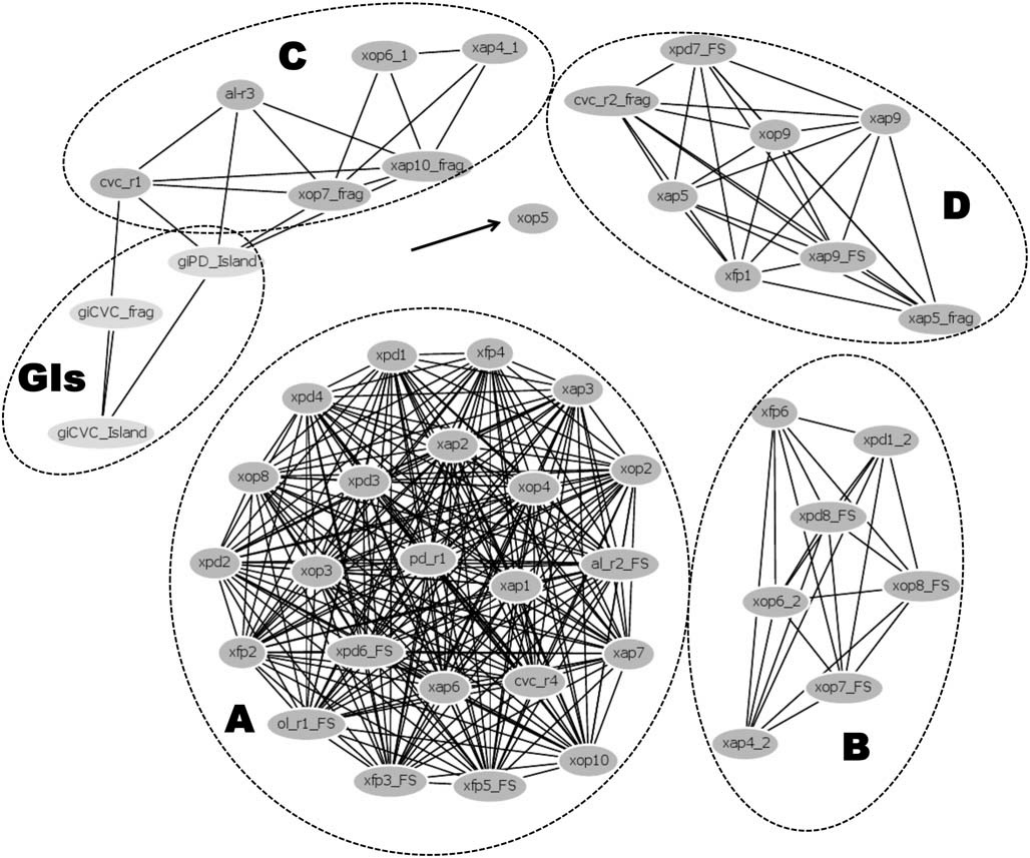
Phage and GI integrases spring-embedded layout incorporating evolutionary distance and BLAST relationship information. Nodes are automatically arranged so that the distance between the proteins reflects their sequence divergence computed by PROTDIST program, and placed into discrete clusters or “containers” corresponding to sub-families, showed by dotted lines and indicated by letters (A–D) for phage integrases and GI for genomic island integrases. The phage integrase xop5, apart from the others integrases, are indicating by a black arrow.

In order to determine the evolutionary relationships among the integrases, protein sequences were organized into clusters (except fragments with less than 100 residues) by pairwise sequence diversity, and are presented in a spring-embedded layout (Figure 4). This network analysis revealed the presence of five clusters of integrases, with one larger cluster consisting of 25 integrases, and four smaller groups with up to eight integrases for each group. This reflects the diversity of the integrases inside each genome and among the four strains analyzed, and reveals distinct evolutionary histories for each group.

**Figure 5:**
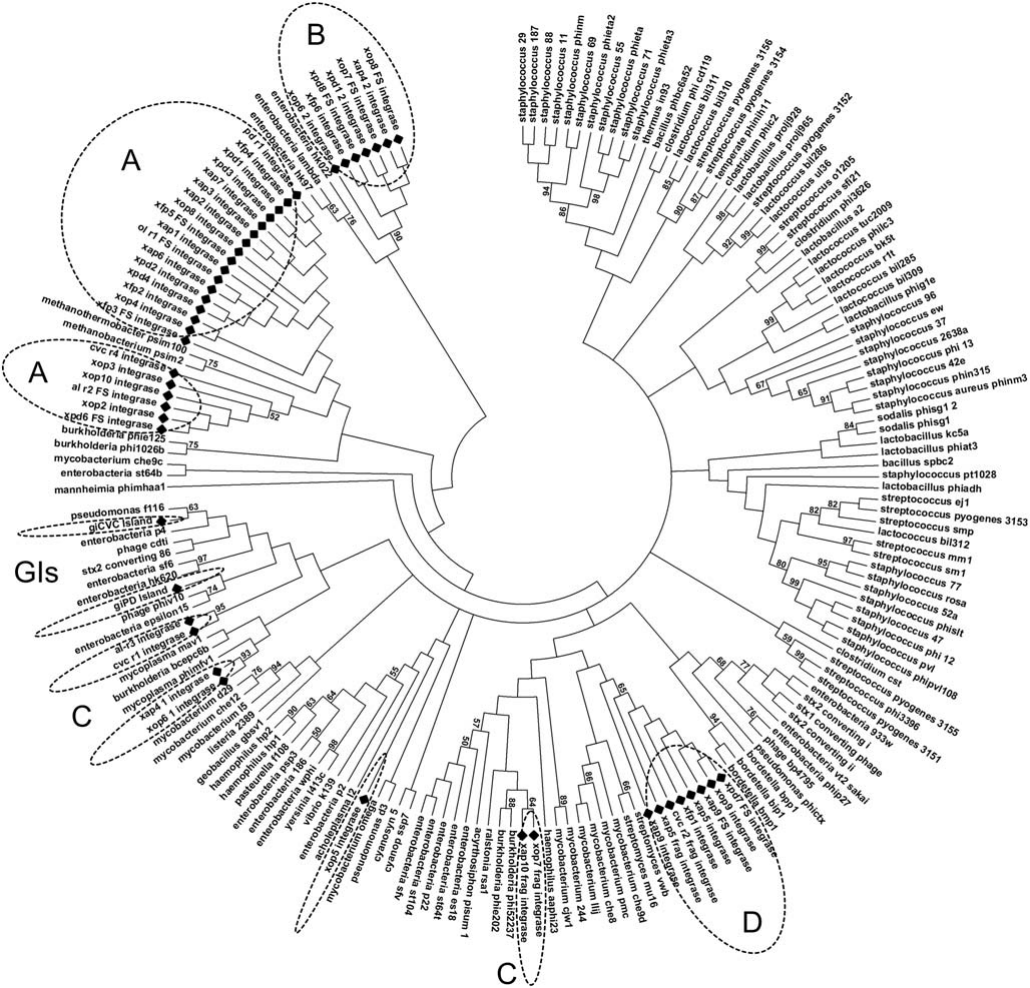
Phylogenetic reconstruction of phage and GI integrases compared to 132 homologues from bacterial phage elements. *Xylella* phage and GI integrases are represented by black diamond-shaped symbols. Distance tree computed by the neighbor-joining method, using the JTT matrix-based method. The dotted lines, and the letters inside, indicate the position of each *Xylella* integrase sub-families (see text for further information).

**Figure 6:**
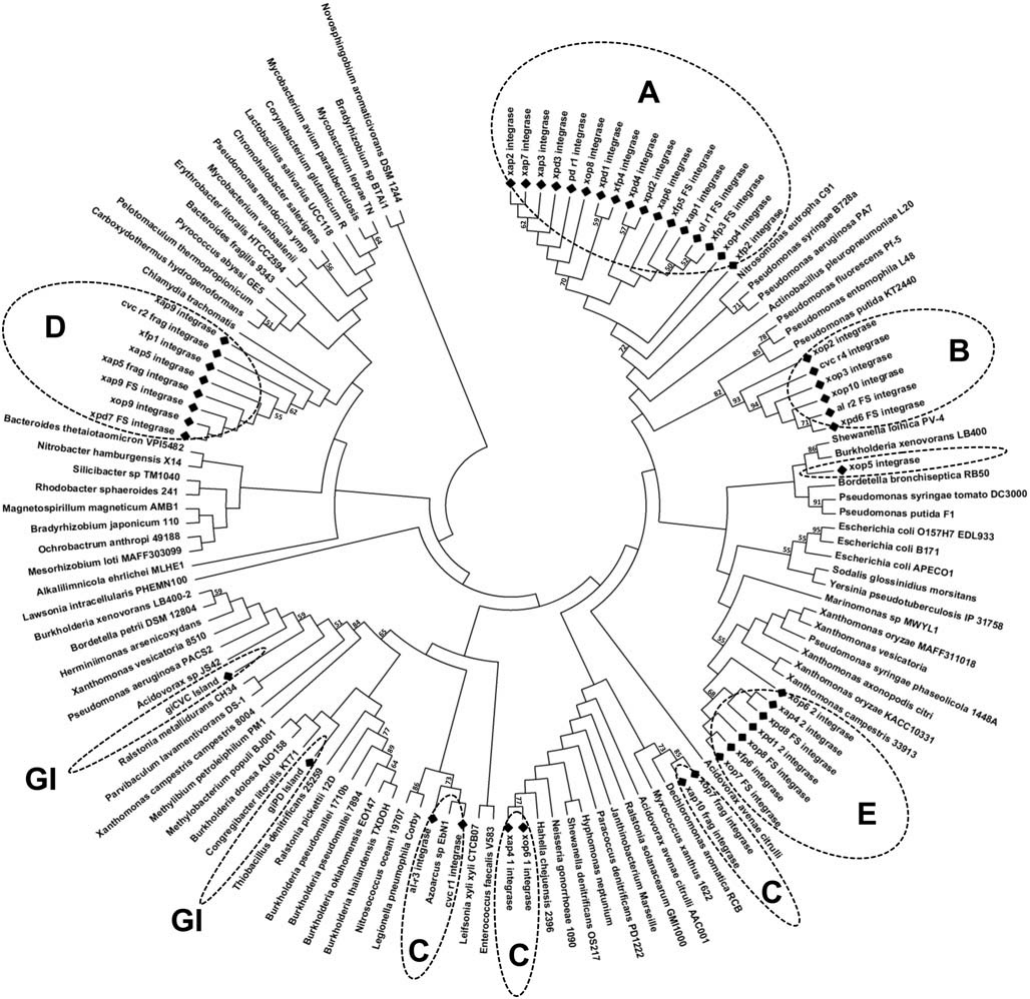
Phylogenetic reconstruction of phage and GI integrases against 186 homologues from viruses. *Xylella* phage and GI integrases are represented by black diamond-shaped symbols. Distance tree computed by the neighbor-joining method, using the JTT matrix-based method. The dotted lines, and the letters inside, indicate the position of each *Xylella* integrase sub-families (see text for further information).

To further evaluate such diversity, the integrase sequences were aligned against 186 integrases present inside phage particles (Figure 5) and against 132 homologues from prophage-like regions of other bacterial species (Figure 6). Interestingly, the two different approaches, phylogenetic analysis and network clustering, reveal the same groups of integrases, denoted in Figures 4, 5 and 6 as groups A, B, C, D and GI (discussed further below), as well as the atypical position of xop5. The xop5 integrase is apart from the others: while all *Xylella* phage integrases share at least 60% of identity, xop5 shares a maximum of 45% of identity with the other ones. The main feature of xop5 prophage is the presence of a Tfp pilus assembly protein in the region related to putative structural phages genes. However, this element does not have any well-characterized structural phage gene. This suggests an ancient and defective prophage element, but exclusively in the Ann1 strain genome.

Integrase group A comprises the largest number of integrases, all of them related to probable complete prophages. This integrase group is related to prophages from few beta- and gamma-proteobacteria species, such as *Pseudomonas*, *Actinobacillus* and *Nitrosomonas* (Figure 6), revealing a restricted phyletic pattern. The same restricted neighborhood is noted in a phylogenetic analysis with integrases from viral particles (Figure 5), being related only to Siphoviridae viruses of *Burkholderia* ssp. (a beta-proteobacterium) and Archaea species (*Methanobacterium* and *Methanothermobacter*).

Group B includes eight integrases, and it is related only to prophages from gamma-proteobacteria species (*Escherichia*, *Yersinia*, *Marinomonas* and several *Xanthomonas species*). This is probably a restricted group of integrases, emerging late in the evolutionary scale (Figure 6). *Xanthomonas* is the closest phylogenetically related group to *Xylella*, but it contains a reduced number of prophages. The close relationship of the integrases in both groups points to an early origin for this specific class of phage-related elements, probably dating from before the speciation of the Xanthomonadales. This group is related to three viruses infecting enterobacteria, all belonging to the lambda-like group of Siphoviridae (Figure 5).

Group D comprises eight integrases (including one from a remnant phage region), and it is related phylogenetically to a broader class of prophages infecting Gram-positive and Proteobacteria species (Figure 6). Interestingly, these integrases are related only to viruses infecting Gram-positive Actinobacteria species (*Mycobacterium* and *Streptomyces*), all from Siphoviridae viruses.

Group C is a heterogeneous group, and it is related to the integrases from genomic islands by network clustering (Figure 4). This heterogeneity is also revealed by the phylogenetic analysis, placing the *Xylella* integrases over a broad range of tree branches. Four integrases (xop7, xap10, xop6-1 and xap4-1) branched together, along with integrases of prophages infecting several species of beta- and gamma-proteobacteria (Figure 6). The other two integrases, belonging to remnant regions (al-r3 and cvc-r1) branch with a wider group that includes the integrases from genomic islands (discussed below). Integrases xop7 and xap10 branch with viruses belonging to Myoviridae (mainly from P2-like group) and Podoviridae (mainly from P22-like group) viruses, while xop6-1 and xap4-1 branch elsewhere, along with GI integrases and al-r3/cvc-r1 (Figure 5).

The integrases within the genomic islands of CVC and PD strains (giCVC and giPD) are related to a large group of integrases from prophages of several beta- and gamma-proteobacteria, including several *Burkholderia* and two *Xanthomonas* species (Figure 6). Moreover, they branch with other integrases from group C along with several Podoviridae P22-like viruses (Figure 5). It was recently reported that the integrases with the CP4 domain (in the related enterobacterial phage P4) potentially has cross-talk activity. It was suggested these might be used as molecular tools to modulate virulence in bacteria [36]. However, there is no evidence to support the cross-talk activity of *X. fastidiosa* GI integrases.

Most of the phages associated with *Xylella* integrases are able to do generalized and specialized transduction, suggesting an import role in the genomic evolution of their hosts. Collectively, these results suggest a broad evolutionary history for each group of integrases identified in the *Xylella* genomes. They are related to several groups of prophages, infecting different groups of bacteria, including the closely related groups of beta- and gamma-proteobacteria, as well distantly related groups, such as Firmicutes and Actinobacteria. There is a large diversity of putative *X. fastidiosa* prophages related to Siphoviridae, Podoviridae or Myoviridae viruses. Moreover, the fact that *X. fastidiosa* prophage-like elements are related to several groups of phages, may be potentially misleading and be due to wrong models of viral taxonomical classification (currently based on features encoded by a minority of their genes), as long debated in the literature. For example, Lawrence and colleagues [37], proposed a different classification that takes into account several parameters (as frequency of genetic exchange, ecological isolation, and periodic selection acting upon each group), which could more accurately represents the biological relationships among the viruses.

#### c) Integrases and the role of tRNAs in integration

We also assessed the association between phage-integrases and presence of tRNAs in the vicinity of prophage regions. Despite the high level of similarity shared by the integrases, most of them cannot share the att sites of other. The integrases may have diverse and unknown possibilities for insertion sites, but in *Xylella* 72% of these sites are associated with tRNAs (Table 1). This agrees with previous reports wherein 75% of recombinases are thought to use tRNAs as attachment sites [38]. There are eight types of tRNAs associated with integrases and prophage-like regions: Arg, Asp, Cis, Gly, Lys, Ser, Thr, Val. Insertion of tRNA-Asn from xfp6 and tRNA-Val from xap9 may be followed by disruption and reconstitution of the bacterial tRNA (as also reported in other genomes by [15]).

There are three types of tRNA fragments (Val, Asn, Lys) associated with prophage elements. These fragments are probably relics of an insertion with disruption of the ancestral tRNA without the reconstitution. With respect to tRNA-Gly, present inside prophage elements cvc-r4, xpd6, xop10 and xap3 and not directly involved as site of insertion, it may be a product of phage-mediated LGT to bacteria. This is supported by comparative analysis showing that at least 81 (19%) of a total of 430 phage genomes analyzed bear a tRNA in their genomes and have no direct viral function (Figure S2). They are, in this way, possibly acquired during transduction events, and brought into the host genome by LGT processes.

It is interesting to note that the largest (in length) prophage-like regions, and probable complete and active prophages, are associated with tRNAs with higher numbers of copies in the chromosome (Arg, Ala, Gly and Ser). This indicates that they are preferred sites of insertion and markers for genome rearrangement of recent phage acquisitions. On another hand, none of the inherently unsuitable tRNAs described previously (Glu, Gln, His, Met, Trp) [15] was used as sites of prophage integration in *Xylella*.

## Discussion

This is the first extensive study showing that the prophage-like elements have a role or function in the process of *X. fastidiosa* genome organization and differentiation. The data presented in this work clearly demonstrate the role of the phages in the diversification and speciation of *X. fastidiosa*, both in a short time-scale promoting local and global rearrangements and activation/inactivation of host genes, and in a large evolutionary time-scale promoting speciation within the group by the acquisition of novel “cargo” components. This is highlighted by the diverse and common insertions for the diverse prophages elements, indicating a differential impact of common and diverse prophages in the *Xylella* genomes. Accordingly, it is still unknown if these prophages are functional or if they represent ancient insertions and are stalled in the bacterial chromosomes. Moreover, there is also evidence supporting that the phage activity perhaps is still in process: (1) higher levels of expression of phage-related genes, including those related to induction of the lytic cycle under stress conditions; and (2) direct observation of putative phage-like particles associated with *Xylella* cells *in vitro* [16] and *in planta* by transmission electron microscopy.

Besides being responsible for abrupt large-scale alterations in the structure and organization of *X. fastidiosa* genomes, the prophages are also capable of carrying some “cargo” genes with function not directly related to the phage propagation. In fact, the no assignment of a known phage function may be solely due to our current lack of knowledge concerning these genes. This is supported by the fact that most of these genes are strain specific, and thus they may be associated to specialization of the phage to the host, suggesting an important role in the generation of new variants of bacterial strains.

On the other hand, *Xylella* integrases are mostly related to lambda-like phage integrases. These phages are widely known as genetic mosaics, and some are able to perform generalized transduction that may confer drastic changes in their hosts. These findings suggest a combined model of evolution to *Xylella* integrases and their elements, in which site specific and illegitimate recombination take place, and the mosaic architecture of the prophage elements represent a creative process in order to generate genetic variation, driving forces to the evolution of this genera.

Taken together, these results helped to determine the role and diversity of each prophage-like region, disclosing the mechanism and integration sites of the integrases associated to these regions and their influence in the differentiation of *Xylella* strains genomes. Also, the data support that phage regions are transcriptionally active and may be producing virus-like particles; however, the association between expression and disease development remains to be demonstrated. Also, whether the enrichment of phage related ORFs is associated with gene duplication or virus-like particle formation is unknown. These observations open avenues to functional studies that may result in alternative strategies of disease control.

## Materials and Methods

### Identification of Phage-related Integrases

Potential ORFs with gene products assigned as integrases were identified by keyword and protein domain searches with BLAST program [39], against the CDD database, and aligned with CLUSTALX 2.0 [40] to further assign the position of the catalytic residues. Phylogenetic trees were constructed using Neighbor Joining (NJ) algorithm [41] and bootstrap assessment (500 replicates) as implemented in PHYLIP package [42]. Trees were edited with MEGA4 program (Molecular Evolutionary Genetics Analysis) [43].

Two-dimensional distance-constrained, spring-embedded and cluster-based phage-integrase network layouts were constructed with InterView program [44], with the phage integrases interactions determined by an all-against-all BlastP program with an e-value of 10^−5^.

### Identification of Prophage-like Regions

All previously described prophages in the Xf-CVC and Xf-PD genomes [1], [3] were used as reference to identify potential prophage regions in Xf-ALS and Xf-OLS genomes by similarity search. The Xf-ALS and Xf-OLS candidate prophage-like regions were computed and automatically annotated with BlastX searches [39]. The resultant annotation was searched by using specific keywords (e.g. phage, integrase, tail, capsid, terminase, portal, head, neck, fiber), and for the positive matches a neighbor analysis was performed in order to find more phage-like genes. A minimum size of 10,000 bp was required to elect any potential prophage region. Smaller regions were individually analyzed and compared to the other genomes' relative positions to define them as prophage remnant regions (rm). The same procedure was used to identify new potential prophage-like regions as well as remnant regions in the Xf-CVC and Xf-PD genomes.

### Functional Annotation of Prophage-like Regions

Functional annotation of ORFs within potential phage regions was carried out by using the SABIA package [45] with identification of phage landmarks such as tRNA vicinity insertion. For putative functional attribution, BLAST searches [39] and COG [46], INTERPRO [47], PRINTS [48], PSORT [49] and SWISSPROT [50] databases were used.

### Clustering of prophage-like ORFs (Phage Navigator Comparative Database)

SABIA Comparative software [51] was employed to identify the common ORFs clusters of prophage regions. This tool was adapted to perform comparative analysis amongst all *X. fastidiosa* prophage-like elements and 402 phage genomes deposited in GenBank (http://www.ncbi.nlm.nih.gov/genomes/static/phg.html)). All the comparative analyses were based on the Bidirectional Best Hit (BBH) methodology [52] with the following parameters: 60% of query coverage and e-value of 10^−5^. Comparisons between the prophage-like full-length elements were carried out with the M-GCAT program [53] and the EMBOSS package [54] both with default values. Comparisons between upstream and downstream regions of each ORF were performed with CLUSTALW 2.0 and custom PERL-scripts (http://www.perl.com), by the methodology proposed by Souza R.C. (unpublished data).

Scripts in PERL and PHP (http://www.php.net) were written in order to generate the prophage gene maps (originally provided by LBI – Laboratory of Bioinformatics of University of Campinas, the web-based navigator to the prophage genes and the database with integrases information). Further information and all supplementary material are available on the project website: http://gracilaria.ib.usp.br/integraseDB.

### Microarray data acquisition and analysis

Meta-analyses of independent microarray datasets were performed in order to study the gene expression pattern of prophage-like elements in CVC strain in different heat shock conditions. Microarray data were extracted from series GSE3044, GSE4161, GSE4960, GSE6619 and GSE8493 [27]–[30] on NCBI's Gene Expression Omnibus (GEO) database (http://www.ncbi.nlm.nih.gov/geo) site [55]. All data processing information is available under request to the authors. Briefly, we established the detection limit for each slide and each dye as the mean plus 3–5 times the standard deviations of negative control spots. Cy5- and Cy3-derived intensity data from the direct comparisons between the test sample and reference sample were corrected for intensity-dependent dye biases using a Lowess function implemented in the R package (The R Project for Statistical Computing [http://www.r-project.org]). Differentially expressed transcripts or differentially gene contents were identified by using the Significance Analysis of Microarray (SAM) method [56] with a false discovery rate (FDR) <2–5%. The data from GSE4161 was obtained direct from the original paper [30] supplementary materials (http://blasto.iq.usp.br/~tkoide/Xylella/Heat_shock).

## Supporting Information

Figure S1 Multiple alignment of *Xylella* phage integrases primary sequence (groups A to E). Alignment done with CLUSTALX 2.0 program and manually adjusted. Red boxes, the conserved catalytic residues. Only part of each alignment, containing the conserved residues, is shown.
(0.15 MB DOC)

Figure S2 Distribution of tRNAs in 81 phage genomes (out of 430 available in the NCBI database) (detection by tRNAscan-SE). Frequency is given in relation to the number total of tRNAs identified in all genomes.
(0.03 MB DOC)
