# Supplementary material for: Origins of the Xylella fastidiosa Prophage-Like Regions and Their Impact in Genome Differentiation
Source: PLoS One. 2008 Dec 31;3(12):e4059. doi: 10.1371/journal.pone.0004059 (PMC2605562; doi:10.1371/journal.pone.0004059)
Supplement: Figure S1 — Multiple alignment of Xylella phage integrases primary sequence (groups A to E). Alignment done with CLUSTALX 2.0 program and manually adjusted. Red boxes, the conserved catalytic residues. Only part of each alignment, containing the conserved residues, is shown. (0.15 MB DOC) [file pone.0004059.s001.doc]

**Figure S1:** Multiple alignment of *Xylella* phage integrases primary sequence (groups A to E). The alignment done by CLUSTALX 2.0 program with manual adjustment. In red boxes, the conserved catalytic residues. Only part of each alignment, containing the conserved residues, is shown.
