# Supplementary material for: Origins of the Xylella fastidiosa Prophage-Like Regions and Their Impact in Genome Differentiation
Source: PLoS One. 2008 Dec 31;3(12):e4059. doi: 10.1371/journal.pone.0004059 (PMC2605562; doi:10.1371/journal.pone.0004059)

**Figure S2:** Distribution of tRNAs in 81 phage genomes (out of 430 available in the NCBI database) (detection by tRNAscan-SE). Frequency is given in relation to the number total of tRNAs identified in all genomes.


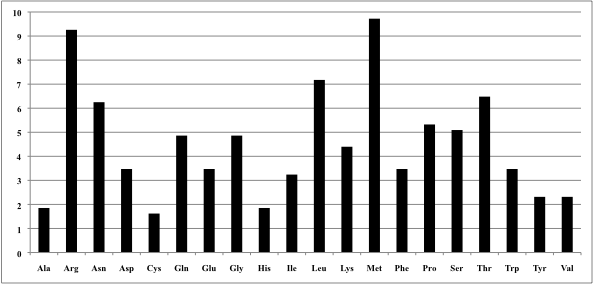

Supplement: Figure S2 — Distribution of tRNAs in 81 phage genomes (out of 430 available in the NCBI database) (detection by tRNAscan-SE). Frequency is given in relation to the number total of tRNAs identified in all genomes. (0.03 MB DOC) [file pone.0004059.s002.doc]
